# Discovery of novel biomarkers for atherosclerotic aortic aneurysm through proteomics-based assessment of disease progression

**DOI:** 10.1038/s41598-020-63229-8

**Published:** 2020-04-14

**Authors:** Hiroaki Yagi, Mitsuhiro Nishigori, Yusuke Murakami, Tsukasa Osaki, Sayaka Muto, Yutaka Iba, Kenji Minatoya, Yoshihiko Ikeda, Hatsue Ishibashi-Ueda, Takayuki Morisaki, Hitoshi Ogino, Hiroshi Tanaka, Hiroaki Sasaki, Hitoshi Matsuda, Naoto Minamino

**Affiliations:** 1 Department of Molecular Pharmacology, National Cerebral and Cardiovascular Center Research Institute, Suita Osaka, Japan; 2 Omics Research Center, National Cerebral and Cardiovascular Center, Suita Osaka, Japan; 3 Department of Vascular Surgery, National Cerebral and Cardiovascular Center, Suita Osaka, Japan; 4 Department of Pathology, National Cerebral and Cardiovascular Center, Suita Osaka, Japan; 5 Department of Bioscience and Genetics, National Cerebral and Cardiovascular Center Research Institute, Suita Osaka, Japan

**Keywords:** Biochemistry, Biomarkers, Diseases, Medical research

## Abstract

Since aortic aneurysms (AAs) are mostly asymptomatic, but they have a high mortality rate upon rupture, their detection and progression evaluation are clinically important issues. To discover diagnostic biomarkers for AA, we performed proteome analysis of aortic media from patients with thoracic atherosclerotic AA (TAAA), comparing protein levels between the aneurysm and normal tissue areas. After hierarchical clustering analysis of the proteome analysis data, tissue samples were classified into three groups, regardless of morphological features. This classification was shown to reflect disease progression stage identified by pathological examination. This proteomics-based staging system enabled us to identify more significantly altered proteins than the morphological classification system. In subsequent data analysis, Niemann-Pick disease type C2 protein (NPC2) and insulin-like growth factor-binding protein 7 (IGFBP7) were selected as novel biomarker candidates for AA and were compared with the previously reported biomarker, thrombospondin 1 (THBS1). Blood concentrations of NPC2 and IGFBP7 were significantly increased, while THBS1 levels were decreased in TAAA and abdominal atherosclerotic AA patients. Receiver operating characteristic analysis of AA patients and healthy controls showed that NPC2 and IGFBP7 have higher specificity and sensitivity than THBS1. Thus, NPC2 and IGFBP7 are promising biomarkers for the detection and progression evaluation of AA.

## Introduction

Aortic aneurysm (AA) is a disease of aortic dilation that results in a bulge or swelling in the aorta. AAs are mostly asymptomatic, but they have a high mortality rate upon dissociation or rupture^1–3^. The early detection and progression evaluation of AA are clinically urgent issues, because surgical treatments, such as the implantation of stent grafts or aortic prosthesis, are available^4,5^. AA is often detected by chance in diagnostic imaging, for example, by computed tomography, magnetic resonance imaging, or ultrasonic echo for other diseases, or during voluntary health examination^6^. However, highly sensitive and specific diagnostic tests for the detection of AA have not yet been developed. D-dimer and C-reactive protein (CRP) levels in the blood have been reported to be suitable for the diagnosis of AA^7–9^. However, these markers have poor disease specificity and effectiveness due to their primary design and aims, and do not reflect aortic tissue degeneration during the formation and progression of the aneurysm^10,11^. Therefore, novel biomarkers based on the molecular mechanism of the development and progression of AA are highly desired for the diagnosis of AA.

A major cause of aortic aneurysm is atherosclerosis. In the advanced atherosclerosis lesion, aortic wall structure is usually disrupted and embrittled. Fragile aortas are then enlarged, resulting in an aneurysm^12^. Although the pathogenic mechanism of atherosclerosis is not fully understood, it is well recognized that endothelial cell dysfunction occurs as a first step, followed by the accumulation of low-density lipoprotein (LDL) in endothelial spaces. LDL is then oxidised and induces inflammation, which stimulates the adhesion of monocytes to endothelial cells and their invasion into the vascular wall. The monocytes then differentiate into macrophages, ingest oxidized LDL, and finally transform into foam cells, which are the hallmark of the initiation of atherosclerosis^13^. During the progression of atherosclerosis, aortic smooth muscle cells (SMCs) differentiate into a synthetic phenotype and actively migrate from the aortic media to the lumenal side^14,15^. SMCs are the main components of atherosclerotic plaques derived from the aortic wall and some of them differentiate into foam cells^16^. Since the degeneration of the aortic media in the atherosclerotic lesion is a crucial step for disease progression, detailed omics analysis of the aortic media would allow the identification of essential factors associated with or participating in the development and progression of AA.

Since blood samples are most frequently used for diagnosis in the clinical setting, due to the minimal invasiveness of their collection, it is preferable to identify biomarkers that are measurable in the plasma or serum. However, the direct identification of biomarkers by comparing blood samples is still challenging, since the high concentrations of abundant proteins prevent the detection of minute alterations in low-concentration proteins. Although previous studies have attempted to identify novel biomarkers for AA in the blood, few biomarkers have been applicable to clinical practice^17–19^. Therefore, we designed this study to search for biomarker candidates from proteins differentially expressed between diseased and normal areas of the aneurysm. This study aimed to rationally select and narrow down candidate biomarkers and determine their blood concentrations in AA patients and controls. Thus, we performed proteomic analysis of diseased and normal areas of the aortic media in the aneurysm tissues excised from patients with thoracic atherosclerotic AA (TAAA). Based on hierarchical clustering analysis of these proteome analysis data, we established a new staging method, designated as a proteomics-based progression staging method, and identified proteins with significantly altered expression levels in AAs. Niemann-Pick disease type C2 protein (NPC2) and insulin-like growth factor-binding protein 7 (IGFBP7) were selected as novel biomarker candidates, and their significant elevation was confirmed in blood samples of patients with TAAA and abdominal atherosclerotic AA (AAAA).

## Results

### Proteome analysis of aortic media from patients with TAAA

As the first step in developing a new diagnostic method for AA, we performed proteome analysis of aortic media from patients with TAAA, to search for novel biomarkers of AA in the aortic wall. Table 1 shows the characteristics of the TAAA patients (n = 29) and non-vascular disease controls (NVDCs, n = 14), and the AAAA patients and healthy controls (HCs) enrolled in this study. Several differences were observed in the clinical indices among TAAA, AAAA, NVDC, and HC subjects enrolled in the present study (See Methods for the indices). Maximal diameter areas (Group D, n = 29) and their proximal areas with normal shape and diameter (Group N, n = 23) in the aneurysm were surgically excised and collected from TAAA patients. Among these tissue samples, six from Group N did not have a sufficient quantity for proteome and pathological analyses, since limited amounts of normal diameter tissues were excised during surgery. Thus, the number of samples in Group N was smaller than the number in Group D. Among the three layers of the aortic wall, i.e., the intima, media, and adventitia, the aortic media can be collected in a relatively uniform quality, since it is separated from the bloodstream and extravascular tissues by the intima and adventitia, respectively. Thus, we immediately collected and stored aortic media samples after separation of the aortic wall for pathological examination.

**Table 1:** Characteristics of patients and controls enrolled in this study.

	TAAA	AAAA	NVDC	HC
Number total (n)	29	51	14	44
Age (yo)	71.1±1.4*^#^	69.1±1.1*^#^	31.7±3.8*	47.4±1.7
Male (n)	27	47	9	24
Female (n)	2	4	5	20
Aortic diameter (mm)	57.0±1.3	52.1±1.1	-	-
Smokers (n)	24	43	5	19
BMI^†^ (kg/m^2^)	23.3±0.6	23.7±0.4	21.0±1.0	22.1±0.4
Systolic blood pressure (mmHg)	122±3^#^	115±2^#^	88±5*	116±2
Diastolic blood pressure^†^ (mmHg)	70±2	64±2	61±4	69±2
TC (mg/dL)	172±6^#^	179±5^#^	130±12*	187±4
LDL-C^†^ (mg/dL)	100±5^#^	108±5^#^	69±9*	102±3
HDL-C (mg/dL)	51±2*	45±2*	40±6*	74±2
TG (mg/dL)	121±14*^#^	152±11*^#^	68±8	64±4
UA (mg/dL)	6.1±0.2*	6.1±0.2*	7.0±0.9*	5.0±0.2
CRP (mg/dL)	0.16±0.04*^#^	0.37±0.10*^#^	4.24±2.06*	0.06±0.03

Data are presented as means ± SEM. †One data in the analysis of the NVDC samples was unknown and excluded from data analysis. Statistical significance (*p* < 0.05) was observed: *compared with the HC, and #compared with the NVDC subjects. TAAA, thoracic atherosclerotic aortic aneurysm; AAAA, abdominal atherosclerotic aortic aneurysm; NVDC, non-vascular disease control; HC, healthy control; BMI, body mass index; TC, total cholesterol; LDL-C, low-density lipoprotein cholesterol; HDL-C, high-density lipoprotein cholesterol; TG, triglycerides; UA, uric acid; CRP, C-reactive protein, yo; years old.

Firstly, to identify differentially expressed proteins between Group D and Group N, we performed non-target proteome analysis using nano-liquid chromatography, coupled with tandem mass spectrometry (LC-MS/MS), followed by quantitative data analysis using the 2DICAL software^20,21^. 2DICAL data analysis resulted in the identification and quantification of 15,240 peptide peaks from all the aortic media samples analysed in this study. Protein levels in the aortic media were then calculated using in-house software, which averaged the multiple tryptic peptide peak data for each protein. Details of the protein identification and quantification procedures are described in the Supplementary Methods. Finally, the expression levels of 1311 proteins in the aortic media of TAAA patients were determined with a high degree of confidence and these were statistically compared.

### Progression staging of TAAA based on proteome analysis data

**Figure 1:**
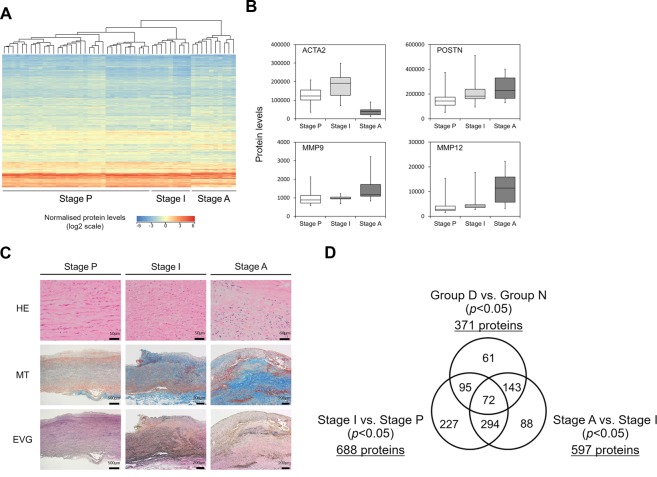
Progression staging of TAAA based on proteome analysis data. (**A**) Dendrogram with heat map generated by hierarchical clustering analysis of proteome analysis data of aortic media of TAAA tissues. Three clusters associated with disease progression were formed and each cluster was later defined as preclinical stage (Stage P), intermediate stage (Stage I), or advanced stage (Stage A), as indicated at the bottom. (**B**) Protein levels of ACTA2, POSTN, MMP9, and MMP12 in the three stages described above are shown as box and whisker plots. Data are expressed as median values and interquartile ranges. (**C**) Histopathological analysis of TAAA tissues at different disease progression stages. Upper panels show HE staining images at a higher magnification, middle panels show MT staining images, and lower panels show EVG staining images. (**D**) Numbers of differentially expressed proteins are shown in Venn diagrams, with comparisons between diseased area (Group D) and normal area (Group N) by the morphological classification system and between Stage I and Stage P and between Stage A and Stage I by the proteomics-based progression staging system. TAAA, thoracic atherosclerotic aortic aneurysm; HE, hematoxylin and eosin; MT, Masson’s trichrome; EVG, elastica-van Gieson.

To determine the differential protein expression profiles between Group D and Group N, hierarchical clustering analysis was performed on proteome analysis data using the Ward’s minimum variance method^22^. Unexpectedly, aortic media tissues of Group D and Group N were clearly classified into three clusters, regardless of their morphological features. Since these clusters were assumed to be associated with the disease progression of TAAA, based on the proteins used for clustering (see below), we tentatively designated the three clusters as Stage P (preclinical), Stage I (intermediate), and Stage A (advanced) (Fig. 1A).

To characterize the three clusters, we compared the expression levels of proteins known to correlate with AA progression in these clusters, based on peak intensities from proteome analysis data (Fig. 1B). The levels of the aortic smooth muscle actin (ACTA2), which is abundantly expressed in aortic and vascular SMCs were higher in Stage I than in Stage P, whereas they were significantly lower in Stage A. This indicated a deficiency of SMCs and ACTA2 in Stage A due to structural remodeling. We also found augmented expression of periostin (POSTN), a matricellular protein promoting tissue fibrosis, as well as matrix metalloproteinase 9 (MMP9) and MMP12, which degrade extracellular matrix components and disrupt tissue organization^23,24^, according to the stage of disease progression (Fig. 1B). These results indicated that the three clusters were associated with the progression of atherosclerotic AA.

To confirm pathological changes in the aortic tissues classified according to proteomics-based clustering, hematoxylin-eosin (HE), Masson’s trichrome (MT), and elastica-van Gieson (EVG) staining were performed using the TAAA tissue sets representing the three tentative progression stages (Fig. 1C and Supplementary Fig. 1). Staining images from these three different methods indicated a loss of SMCs, fibrosis, and elastic fiber fragmentation occurring in the order of the tentative progression staging of TAAA. Taken together, the results of the hierarchical clustering, based on proteome analysis data of the aortic media of TAAA patients, correctly reflected the pathological progression stages and the changes in the expression levels of known proteins associated with atherosclerotic AA. Therefore, the proteomics-based staging was valid for use in the identification of new biomarker candidates.

Next, we compared the numbers of differentially expressed proteins according to conventional morphological classification groups (Groups N and D) with those differentially expressed according to the novel proteomics-based progression stages (Stages P, I, and A) (Fig. 1D). For the morphological classifications, 371 proteins showed significantly different expression levels between Group N and Group D (*p* < 0.05), while for the proteomics-based progression stages, a total of 919 proteins showed significantly different expression levels between Stage I and Stage P (688 proteins) (*p* < 0.05) or between Stage I and Stage A (597 proteins) (*p* < 0.05). We identified 2.5 times more differentially expressed proteins in the aortic media of TAAA patients using the new staging method. Therefore, using this staging method results in an increased chance of identifying new biomarker candidates.

### Selection of candidate biomarkers for AA from the differentially expressed proteins

**Figure 2:**
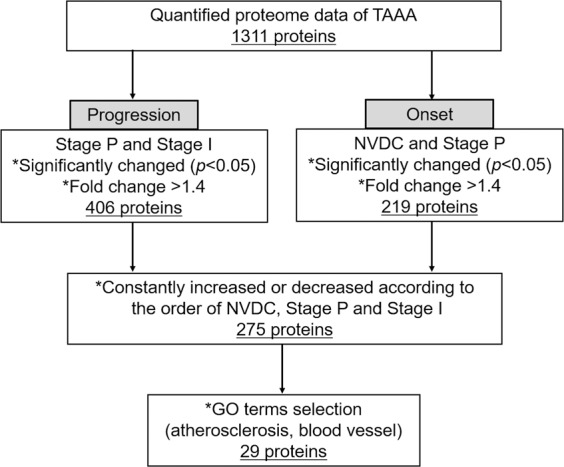
Flowchart of biomarker candidate selection using proteomics-based progression staging. The criteria for selection (asterisks) and the number of proteins narrowed down (underlined) are indicated for each step.

To discover useful and reliable biomarkers, we searched extensively for biomarker candidates for detecting the onset and progression of TAAA from the differentially expressed proteins in the aortic media of TAAA, according to the proteomics-based progression staging. The outline for the selection and investigation of biomarker candidates is shown in Fig. 2. In this selection process, we recognized that the proteins altered between Stage A and other stages were not suitable for selection and thus, they were excluded from the following comparisons. This is because the proteome profile at this stage was greatly different from the profiles of the other two stages (Stages P and I) and marked degeneration and remodeling of the aortic wall were also observed at this stage by histological examination.

First, all proteins differentially expressed between Stage P and Stage I were listed. We then narrowed down from 688 proteins to 406 proteins by selecting those showing more than 1.4-fold change. These proteins were presumed to be associated with the progression of atherosclerotic AA. Next, we focused on the onset of atherosclerotic AA and compared protein levels in Stage P with those of NVDC tissues. Since the tissues in Stage P were in the initial stage of lesion formation, the differentially expressed proteins between Stage P and NVDC were assumed to be associated with the onset of atherosclerotic AA. For the NVDC samples, aortic roots excised from transplant recipients and ascending or descending aorta samples from autopsy cases were collected. Protein expression levels in the aortic media of these samples were analysed by the same proteome analysis method (n = 14, Table 1). Between Stage P and NVDC, 219 proteins showed significantly different expression levels. From the proteins in these two comparisons (total of 543 proteins), we selected those showing an increasing or decreasing trend during the progression of TAAA, i.e., in the order of NVDC to Stage P to Stage I. After this step, 275 proteins remained for selection. The expression levels of the selected proteins were either increased or decreased according to the degree of disease progression and were significantly altered in either the onset (NVDC vs Stage P) or progression (Stage P vs Stage I) processes of TAAA.

To further narrow down the candidate proteins, we performed gene ontology (GO) analysis of the 275 proteins described above. A total of 125 GO terms (false discovery rate <0.05) were enriched and of these, 26 GO terms were associated with atherosclerosis or blood vessel function (Supplementary Table 1). This step further narrowed the selection down to 29 candidates. Table 2 summarises the protein biomarker candidate data, including the fold-changes in the NVDC vs. those in the Stage P or the changes in the Stage P vs. those in the Stage I, and the coefficients of variation (%) for the candidate protein levels in each stage of progression. These candidates included serum paraoxonase/arylesterase 1 (PON1), clusterin (CLU), tumor necrosis factor ligand superfamily member 13 (TNFSF13), lipopolysaccharide-binding protein (LBP), and thrombospondin 1 (THBS1), which have previously been reported as biomarkers of atherosclerosis or cardiovascular disease^25–29^. In addition, five apolipoproteins and two collagens, which are well known to be associated with atherosclerotic AA, were also included in the list, whereas the other 17 proteins had no substantial report definitively describing their association with AA. Therefore, these 17 proteins were likely to be novel biomarker candidates. Among them, we finally focused on NPC2 and IGFBP7, since these were small secretory proteins with augmented expression in aneurysmal tissues, having a high possibility of being released into the bloodstream. Therefore, these two proteins were subjected to subsequent confirmation and validation experiments to determine their suitability as biomarkers of atherosclerotic AA. In addition, we subjected THBS1 to subsequent analysis as a reference among the five reported markers, since this protein was also selected using the morphological classification method (data not shown)^30^.

**Table 2:** Biomarker candidates selected by gene ontology terms associated with atherosclerosis and blood vessel function.

Gene symbol	Secretory	Fold change		Protein name	CV (%)		
		Stage P /NVDC	Stage I /Stage P		NVDC	Stage P	Stage I
ANXA1	-	1.88	1.71	Annexin A1	33	43	13
ANXA2	+	1.78	1.53	Annexin A2	24	32	35
APOA1	+	4.02	1.41	Apolipoprotein A-I	45	92	33
APOA2	+	2.74	1.23	Apolipoprotein A-II	50	66	38
APOC1	+	3.03	1.38	Apolipoprotein C-I	74	53	22
APOC3	+	1.13	1.83	Apolipoprotein C-III	31	35	18
APOE	+	1.64	1.61	Apolipoprotein E	35	50	33
ATP1A1	-	1.01	1.77	Sodium/potassium-transporting ATPase subunit alpha-1	19	25	18
ATP5A1	-	1.13	1.41	ATP synthase subunit alpha, mitochondrial	59	48	34
CD44	-	1.87	1.23	CD44 antigen	26	50	11
CDH13	-	1.38	1.67	Cadherin-13	40	37	20
CLU	+	1.50	1.59	Clusterin	25	42	27
COL15A1	+	1.42	1.09	Collagen alpha-1 (XV) chain	29	38	19
COL1A2	+	1.05	1.67	Collagen alpha-2 (I) chain	32	40	46
GNA13	-	0.42	0.79	Guanine nucleotide-binding protein subunit alpha-13	44	34	21
HEXB	-	1.27	5.42	Beta-hexosaminidase subunit beta	12	36	96
IGFBP7	+	1.46	1.23	Insulin-like growth factor-binding protein 7	61	51	26
LBP	+	1.17	1.85	Lipopolysaccharide-binding protein	27	55	39
NPC2	+	1.02	1.46	Epididymal secretory protein E1	33	50	32
PLCD1	+	1.02	1.41	1-Phosphatidylinositol 4,5-bisphosphate phosphodiesterase delta-1	13	34	30
PON1	+	0.95	0.48	Serum paraoxonase/arylesterase 1	36	69	50
RBP4	+	0.67	0.66	Retinol-binding protein 4	32	44	57
SCP2	-	1.18	1.48	Non-specific lipid-transfer protein	26	41	35
SEMA3C	+	1.51	1.14	Semaphorin-3C	40	46	33
SOD1	-	1.18	1.45	Superoxide dismutase [Cu-Zn]	28	42	35
TGM2	+	1.56	1.67	Protein-glutamine gamma-glutamyltransferase 2	36	47	38
THBS1	+	1.13	1.48	Thrombospondin-1	32	39	19
THY1	-	1.99	1.28	Thy-1 membrane glycoprotein	20	42	44
TNFSF13	+	2.09	1.22	Tumor necrosis factor ligand superfamily member 13	46	42	38

NVDC, non-vascular disease control; CV, the coefficient of variance of protein levels in each stage.

### Confirmation of expression of biomarker candidates in atherosclerotic AA

**Figure 3:**
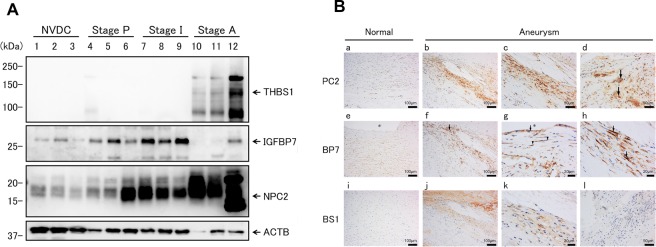
Expression and localization of NPC2, IGFBP7, and THBS1 in aortic tissues. (**A**) Protein levels of THBS1, IGFBP7, and NPC2 in aortic tissues of NVDCs and those of three different stages (Stages A, B, and C) of TAAA patients were determined by western blotting analysis. ACTB was used as an internal control. Arrows indicate the predicted positions of THBS1 (129 kDa), IGFBP7 (29 kDa), NPC2 (17 kDa), and ACTB (42 kDa), respectively. Whole gel images are presented in Supplementary Fig. 2. (**B**) Immunohistochemical analyses of NPC2, IGFBP7, and THBS1 (first, second, and third row, respectively); Staining data for normal aortas of NVDCs are shown in a, e, and i of the left column, while those of TAAAs are shown in b-d of the first, f-h of the second, and j-l of the third rows. Arrows in d indicate NPC2-positive foam cells. Arrows in f, g, and h indicate IGFBP7-positive foam cells, endothelial cells, and smooth muscle cells, respectively. Arrowheads in g indicate IGFBP7-positive smooth muscle cells. The asterisks in e and g indicate the lumenal region. NVDC, non-vascular disease control; TAAA, thoracic atherosclerotic aortic aneurysm.

To confirm the expression of NPC2, IGFBP7, and THBS1 in aortic media and their changes according to progression stages, we performed western blotting analysis using antibodies specific for each protein (Fig. 3A and Supplementary Fig. 2). The expression level of NPC2 increased in the order of disease stage progression, with especially high levels of expression detected in Stage A. IGFBP7 levels were elevated in a similar manner, except that its levels clearly decreased at Stage A. THBS1 was only detected at Stage A. Although aliquots of equivalent tissue lysates were loaded, the levels of β-actin (ACTB) and glyceraldehyde-3-phosphate dehydrogenase (GAPDH), commonly used as internal controls, were decreased in the order of progression stages, especially in the Stage A, that was mainly induced by tissue remodeling, replacement of SMCs by connective tissues. Even after normalisation by ACTB or GAPDH levels, the protein levels of NPC2, IGFBP7, and THBS1 were elevated according to the progression stage. Apparent differences in the band intensity in Stage A were derived from the tissue remodeling that occurred to a different extent in each patient. In the THBS1 and NPC2 lanes, extra bands were observed in addition to the predicted molecular size bands. Higher molecular weight bands have been recognized as the glycosylated forms of THBS1 and NPC2, since each has several glycosylation sites that have been reported to be glycosylated^31,32^. Lower molecular weight bands were probably the proteolytic products of THBS1 and NPC2, since proteolysis in the aortic media has been known to be accelerated by activated proteases, such as MMP-9, in the atherosclerotic AA region of TAAA.

Immunohistochemical staining was performed to further evaluate the expression and localization of NPC2, IGFBP7, and THBS1 in normal and aneurysmal aortic walls. In the normal aorta, NPC2 and IGFBP7 were weakly positive in SMCs, but THBS1 was negative (Fig. 3B [a, e, and i]). In the aortas of TAAA patients, NPC2 and IGFBP7 were expressed mainly in the atheroma, eroded atheroma, and residual media (Supplementary Fig. 3). THBS1 was mainly expressed in the thrombus and atheroma (Supplementary Fig. 3). NPC2 and IGFBP7 were highly expressed in SMCs (Fig. 3B[b,c,h]) and foam cells (Fig. 3B[d,f]), located around cholesterol clefts. IGFBP7 was also expressed in endothelial cells (ECs) and SMCs in the intima (Fig. 3B[g]). THBS1 was expressed in the intima and in the SMCs of the media, located around cholesterol clefts (Fig. 3B[j,k]), whereas it was not expressed in foam cells (Fig. 3B[l]). In agreement with the proteome analysis data, the expression levels of these proteins were elevated in the aortic wall of TAAA patients compared with the normal aortic wall of NVDCs (Fig. 3 and Supplementary Fig. 3). However, the expression and localization of these proteins within the aortic wall differed from each other, especially between THBS1 and the other proteins. This indicated that the alteration of these proteins may reflect different aspects of atherosclerotic AA.

### Measurement of biomarker candidates in blood samples of TAAA and AAAA patients and HCs

**Figure 4:**
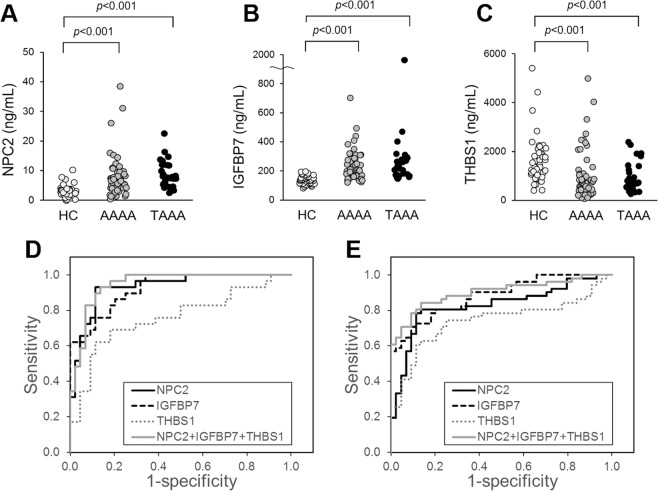
Concentrations of NPC2, IGFBP7, and THBS1 in blood samples of HCs and patients with AAAA and TAAA. The concentrations of (**A**) NPC2 in serum, (**B**) IGFBP7 in serum, and (**C**) THBS1 in plasma of 44 HCs, 51 AAAA patients, and 29 TAAA patients were measured using an ELISA kit specific for each protein. (**D**) ROC analysis of NPC2 (black line), IGFBP7 (black broken line), THBS1 (grey dotted line), or the combination of all three proteins (grey line) in HCs and TAAA patients. The AUCs were 0.933, 0.925, 0.759, and 0.948 for NPC2, IGFBP7, THBS1, and their combination, respectively. (**E**) ROC analysis of NPC2 (black line), IGFBP7 (black broken line), THBS1 (grey dotted line), or the combination of all three proteins (grey line) between HCs and AAAA patients. The AUCs were 0.828, 0.882, 0.742, and 0.901 for NPC2, IGFBP7, THBS1, and their combination, respectively. HC, healthy control; AAAA, abdominal atherosclerotic aortic aneurysm; TAAA, thoracic atherosclerotic aortic aneurysm; ROC, receiver operating characteristic; AUC, area under the curve.

To determine whether alterations in these protein levels in the aortic media reflected their respective blood concentrations, we measured the concentration of NPC2, IGFBP7, and THBS1 in the blood samples of patients with TAAA (n = 29) and AAAA (n = 51) and HC subjects (n = 44), whose characteristics are summarised in Table 1. NPC2 and IGFBP7 levels were measured in serum, while THBS1 was measured in plasma, using commercially available enzyme-linked immunosorbent assay (ELISA) kits. NPC2 and IGFBP7 concentrations were significantly elevated in patients with TAAA and AAAA (for either protein, TAAA: *p* < 0.001 and AAAA: *p* < 0.001, Steel-Dwass test) compared with HC subjects (Figs. 4A,B). In contrast, THBS1 concentration was significantly lower in patients with TAAA and AAAA (TAAA: *p* < 0.001, AAAA: *p* < 0.001, Steel-Dwass test) than in HC subjects (Fig. 4C). We performed receiver operating characteristic (ROC) analysis of the three proteins between the TAAA and HC groups. The areas under the curve (AUCs) obtained from the ROC analysis of NPC2, IGFBP7, and THBS1 were 0.933, 0.925, and 0.759, respectively (Fig. 4D). We also performed ROC analysis between the AAAA and HC groups. The AUCs for NPC2, IGFBP7, and THBS1 were 0.828, 0.882, and 0.742, respectively (Fig. 4E). These results demonstrated that NPC2 and IGFBP7 were biomarker candidates that were clinically useful for the diagnosis of both TAAA and AAAA, but especially for TAAA. Moreover, they were superior to the previously reported biomarker, THBS1. Furthermore, the combined AUCs for these three proteins were 0.948 and 0.901 for TAAA and AAAA, respectively, indicating their improved performance as biomarkers when used in combination. Taken together, the present results showed the highly improved performance of NPC2 and IGFBP7 as biomarkers for the detection of AA, when compared to previous biomarkers, especially in the case of TAAA.

## Discussion

To discover novel biomarkers for the detection and progression evaluation of atherosclerotic AA, we performed proteome analysis of aortic media from TAAA patients and NVDCs. The aortic media is mainly composed of SMCs and matrices within the aortic wall and are recognized as the target tissue for the initiation and progression of atherosclerosis, which often results in AA. Therefore, the proteome analysis of aortic media of TAAA patients with different stages of disease progression was expected to identify biomarker candidates applicable to clinical practice. Using a morphological classification system, aortic aneurysm tissues were divided into aneurysm (Group D), border, and normal areas (Group N) based on their diameter and pathological features. Unexpectedly however, after hierarchical clustering analysis of proteome analysis data from the aortic media of TAAA patients (Groups D and N), aortic media samples were classified into three clusters according to their disease progression stage. This classification was confirmed by pathological examination, as well as by measuring the expression levels of proteins known to be altered in atherosclerotic AA (Fig. 1A-C). Of the 29 samples in Group D, 13 samples were categorized as Stage P and 10 samples as Stage A, using the new proteomics-based staging system (Supplementary Table 2). This indicated that aortic media from the initial to advanced lesion stages were included in Group D, resulting in fewer differentially expressed proteins detected using the conventional morphological classification system. By introducing the proteomics-based progression staging system (Fig. 1D), we successfully identified 2.5 times more differentially expressed proteins, which is advantageous not only in the search for novel biomarkers, but also in elucidating the mechanisms involved in the pathogenesis and progression of TAAA.

To select useful and reliable biomarker candidates for atherosclerotic AA, we identified differentially expressed proteins using the proteomics-based progression staging system and then narrowed down the selection using several criteria, as shown in Fig. 2. Since sufficient numbers of the final candidates were not obtained, we reduced the threshold of the fold change to>1.4 and found 29 candidate proteins, which have been listed in Table 2. In this list, two novel biomarker candidates, NPC2 and IGFBP7, were identified along with several proteins, such as THBS1, that have been reported to be involved in atherosclerosis or AA.

One of the identified candidates was NPC2, a small secretory protein of 131 residues, also known as NPC intracellular cholesterol transporter 2^32–34^. Cholesterol accumulation is a crucial event in the development of atherosclerosis, and physiological roles of NPC1 in cholesterol transport and its association with atherosclerosis have been reported^35^. Although NPC2 is known to bind cholesterol and promote cholesterol transport, in conjunction with NPC1, its contribution to atherosclerosis has not yet been established. In atherosclerotic plaques, lysosomal enzymes are released from macrophages and hydrolyze LDL^36^. NPC2 is presumed to participate with NPC1 in cholesterol transport in lysosomes. From these data, it is reasonable to hypothesize that NPC2 is actively expressed in and secreted from macrophages, accumulates in atherosclerotic plaques, and shows elevated blood concentrations. In parallel with this hypothesis, immunohistochemical staining of NPC2 in atherosclerotic AA showed strong positive signals in the atheroma, in foam cells around cholesterol clefts, and in SMCs (Fig. 3B).

IGFBP7 is a 256-reside secretory protein that is expressed in many tissues and is known to be involved in various cellular functions, such as angiogenesis, cellular senescence, and apoptosis^37^. Although the elevated circulating IGFBP7 levels have been reported in heart failure, chronic obstructive pulmonary disease, and soft tissue sarcoma^38–40^, no relationship has been identified between IGFBP7 and atherosclerosis or AA. In the present study, IGFBF7 protein levels were already found to be augmented in Stage P and Stage I (Fig. 3A). Immunohistochemical staining showed that IGFBP7 was mainly present in the atheroma, SMCs, and foam cells around cholesterol clefts (Fig. 3B). Taking these data together, we proposed that NPC2 and IGFBP7 were clinically applicable biomarkers, since these two proteins were actively expressed in foam cells and SMCs in the atherosclerotic lesions of AA patients. Moreover, their expression levels changed according to the stage of disease progression, resulting in elevated blood concentrations via their secretion into the circulation.

THBS1 is a large glycosylated secretory protein of 1152 residues, and has adhesive properties towards extracellular matrices, such as fibrinogen, collagen, and fibronectin. This protein has previously been reported as a biomarker of AA^25,30,41^, was not expressed in foam cells, but was highly expressed in the intima, media, and thrombus of TAAAs (Fig. 3B and Supplementary Fig. 3), in addition to platelets and most tissues, such as the liver and the lungs. The protein level of THBS1 was especially elevated at Stage A in a manner distinct from those of NPC2 and IGFBP7 (Fig. 3A), suggesting that THBS1 expression may reflect pathological conditions different from those of NPC2 and IGFBP7. On the contrary, plasma THBS1 levels were decreased in the TAAA and AAAA groups compared with the HC group (Fig. 4C). The causes of discordance between the tissue and plasma levels of THBS1 in TAAA patients remained unclear, its high expression level in platelets and in the inner side of the aortic wall and the thrombus may be related with the discorded results.

Moxon *et al*. first identified THBS1 as a marker of the abdominal AA by proteomic analysis of the intra-arterial thrombus secretions^25^. In this report, the serum THBS1 levels were also decreased in patients with the abdominal AA (*p* = 0.002), though the difference was rather small; the median of THBS1 levels was 6% lower in the AA group than in the non-AA control group. In our present study, the median plasma THBS1 level in the AAAA group was 41% lower than that of the HC group (*p* < 0.001). Although the concentration range was approximately 30-fold lower in our study, probably because of the difference in samples (serum and plasma), a significant decrease in the THBS1 levels was commonly observed in both the studies.

The blood levels of NPC2 and IGFBP7 in the TAAA groups were two fold-three fold higher than those in the HC group (Fig. 4A), which was greater than the observed changes (1.02 fold-1.46 fold) in the proteome analysis data (Table 2). Since both NPC2 and IGFBP7 proteins have rigid three-dimensional structures formed by multiple disulphide bonds, their proteolytic digestion was probably hindered and the efficiency in the identification and quantification of tryptic peptides had probably declined. These factors have been suggested to result in the reduced fold changes of NPC2 and IGFBP7 between the progression stages in the proteome analysis.

ROC analysis showed that NPC2 and IGFBP7 had higher diagnostic ability for TAAA and AAAA than the known AA biomarkers, based on the data shown in Fig. 4 (THBS1) and based on the previously reported data (MMP-9, C-C motif chemokine 20, myosin heavy chain 11, and Galectin-3)^42–45^. We also analysed the correlation between the blood levels of NPC2, IGFBP7, and THBS1 with those of LDL cholesterol (LDL-C), high-density lipoprotein cholesterol (HDL-C), total cholesterol (TC), triglyceride (TG), and CRP. There was no significant correlation between these indices in patients with TAAA and AAAA. Even when HC subjects were added to the group of AA patients, a weak negative correlation (r = −0.36) was observed only between the NPC2 and the LDL-C levels and not between other combinations. Collectively, NPC2 and IGFBP7 have been considered as diagnostic biomarker candidates that are superior to the known AA biomarkers like THBS1 and have practically no correlation with the blood indices of lipid metabolism and inflammation.

Based on the data described above, we concluded that these two proteins are promising biomarkers that should be clinically validated by measuring their levels in different stages and types of AA. THBS1 also showed diagnostic utility, but at a lower extent than NPC2 and IGFBP7. Since there are no atherosclerotic AA biomarkers in clinical use that directly reflect the pathological conditions of AA tissue, the newly identified biomarkers, NPC2 and IGFBP7, are expected to be effective in clinical applications. By evaluating correlation of their blood concentrations with clinical data of patients with AA and other related diseases in the large-scale study, the clinical significance of these biomarkers will be elucidated. This will pave the way for the diagnostic use of these biomarkers in clinical settings.

There were several limitations of this study. Firstly, this study was a single-centre investigation with a limited number of patients and subjects; i.e., the numbers of TAAA, AAA, NVDC, and HC subjects were 29, 51, 14, and 44, respectively. In particular, the number of NVDCs was limited, due to the reasons described below. Thus, the blood levels of these novel biomarkers have not been compared in patients with different progression stages. Secondly, there was a large age difference between patients with TAAA or AAAA and NVDCs. Among the 14 NVDC samples collected, nine samples were from the root portions of aortas accompanying hearts excised from transplant recipients. Thus, the main reason for the age difference was the restriction on who can receive heart transplantations in Japan. We also collected additional aorta samples from autopsy cases who had not been diagnosed with vascular disease. These were in an age range comparable to TAAA and AAAA patients. However, pathological examination of these samples showed that most of the aortic tissues had atherosclerotic denaturation and only five samples were judged to be usable as NVDCs. There were also age differences between patients with TAAA or AAAA and HCs, since HC blood samples were collected during medical examinations of the general population. Thirdly, there was an unexpected personal and regional heterogeneity of the collected aortic tissues and the two sample areas (normal and diseased) were not uniformly dissected and collected from AA tissues. Fourthly, our proteome analysis only targeted the aortic media of AAs and other aortic wall components, such as the intima, adventitia, and endothelium, have not yet been analysed. Finally, we did not confirm the genetic background of the TAAA and AAAA patients. Therefore, these data need to be interpreted and discussed under these limitations.

In conclusion, we established a novel progression staging system for TAAA on the basis of the protein expression profiles in the aortic media, which consisted of the preclinical, intermediate, and advanced stages, and were confirmed by pathological evaluation. NPC2 and IGFBP7, the two novel biomarkers for TAAA and AAAA, would have not been discovered without this progression staging. This demonstrates that the proteomics-based progression staging system is effective and innovative as compared with the conventional morphological classification used in most studies. The present study will contribute towards establishing proteomics-based disease progression staging systems in other diseases, and may facilitate the discovery of new biomarkers.

## Methods

### Characteristics of the enrolled subjects

This study followed the principles of the Declaration of Helsinki and all patients provided written informed consent for tissue sampling and proteome analyses. The protocol was approved by the Institutional Review Board of the National Cerebral and Cardiovascular Center, Japan (M22–029). Aortic wall tissues were collected from TAAA (n = 29) and NVDC (n = 14) patients. Blood samples were collected from TAAA (n = 29) and AAAA (n = 51) patients, and HC volunteers (n = 44), whose biochemical test results were within the normal limits. Details of the characteristics of the enrolled subjects have been listed in Table 1.

NVDC patients were younger than the AA group patients (TAAA and AAAA). Among 14 NVDC patients, nine patients were heart transplant recipients, where the recipients were restricted by age in Japan, while the others were autopsy cases. HC subjects were also younger than the AA patients, since HC blood samples were collected during medical examinations of the general population. Mainly due to these reasons, there were age differences between the TAAA or the AAAA patients and the NVDC or the HC subjects. Levels of TC, LDL-C, and TG, and the systolic blood pressure were higher in the AA group than those in the NVDC patients. Levels of UA and TG were higher, and HDL-C were lower in the AA group than those in the HC subjects. Only in the case of CRP, the NVDC patients showed higher values than the AA group and the HC subjects, probably due to the implantation of a ventricular-assisted device (transplantation) and the courses of treatment (autopsy).

### Tissue collection

Aortic wall tissues from the patients with TAAA were collected during elective surgery. NVDC thoracic aortic tissues were collected from the ascending or descending aortas of autopsy cases or from the root portions of aortas accompanying hearts excised from transplant recipients. The NVDC tissues had no prominent symptoms of atherosclerosis or vascular disease.

Aortic wall tissues with dissociation or calcification were excluded to maintain the homogeneity of the tissues analysed. Aortic tissues were then divided into two sections according to the degree of morphological disease progression, as determined by the surgeons: 1) diseased area - maximum diameter region of the AA and 2) normal area - normal diameter and shape of the aorta. The border area between the normal area and the diseased area in the aneurysm was removed. Each tissue was further dissected into the intima, media, and adventitia. In this study, aortic media of the diseased area and the normal area from the TAAA patients were used for proteome analysis. These aortic media were weighed, divided into pieces, and frozen in liquid nitrogen. Aortic media of the NVDC tissues were also prepared using the same procedures. All samples were stored at −80 °C until proteome analysis.

### Blood sample preparation

Blood samples were collected from the TAAA patients and the AAAA patients immediately before surgery, and not from the NVDC patients. Blood samples were also collected from the HC volunteers. Serum and plasma were separated by one or two centrifugation steps, respectively, at 3,000 rpm for 15 min at 4 °C. All samples were stored at −80 °C until biomarker measurements.

### LC-MS/MS analysis and protein identification and quantification

Tissue digestion and MS analysis were performed according to our previous report^21^. Aortic media tissues were pulverized under frozen conditions at −80 °C using a Shake Master Neo instrument (BMS, Tokyo, Japan), suspended in methanol, and aliquoted to obtain a target amount of tissue (2 mg) in each tube. After centrifugation and evaporation to dryness, the pulverized tissue was suspended in 2% sodium deoxycholate and heated at 95 °C for 10 min to denature proteins and inactivate intrinsic proteases. After cooling, urea (final concentration: 2 M) and ammonium bicarbonate (final concentration: 50 mM) were added and denatured tissues were digested with lysyl endopeptidase (1 μg; WAKO Pure Chemicals, Osaka, Japan) at 37 °C for 3 h, followed by trypsin (3.3 μg; sequence grade; Promega, Madison, WI, USA) at 37 °C for 20 h. Sodium deoxycholate was removed using the phase-transfer method^46^, and the resulting digests were desalted using C18 StageTips^21,47^. Tryptic peptides were dissolved in 50 μL of 0.1% formic acid and their concentrations were determined using a Qubit fluorometer (Life Technologies, Carlsbad, CA, USA). A portion of the tryptic peptide fraction (300 ng) was separated using a NanoFrontier nano-LC instrument (Hitachi High-Technologies, Tokyo, Japan), with a Halo ES-C18 column (0.2 × 50 mm, 2.7 μm, 160 Å; Michrom Bioresources, Auburn, CA, USA), using a gradient elution of acetonitrile in 0.1% formic acid from 2.0 to 11.6% acetonitrile for 5 min, followed by 11.6 to 45.2% acetonitrile for 60 min. The column effluent was introduced to a TripleTOF 5600 tandem mass spectrometer (AB SCIEX, Framingham, MA, USA) and subjected to high-resolution MS and MS/MS analyses. Peptides were identified using MASCOT software (version 2.4.1; Matrix Science, London, UK), using UniProt as the reference database. Quantitative estimation of peptides was performed using 2DICAL2 software (version 1.3.16; Mitsui Knowledge Industry, Tokyo, Japan)^20,21^. Then, the protein levels were calculated using multiple peptide data from each protein. The detailed procedure for identification and quantification of proteins is summarised in Supplementary Methods.

### Proteome data analysis

Hierarchical cluster analysis of proteome data was performed with GeneSpring software (Agilent Technologies, Santa Clara, CA, USA), using Ward’s method. Differentially expressed proteins were analysed using the Database for Annotation, Visualization, and Integrated Discovery tool (http://david.abcc.ncifcrf.gov/) to calculate the enrichment of GO terms in biological processes^48,49^.

### Western blotting analysis

Approximately 2 mg of pulverized aortic media was homogenized and solubilized in 300 μL of 60 mM Tris-HCl buffer (pH 6.8; containing 2% sodium dodecyl sulfate [SDS], 5% glycerol, and 1% 2-mercaptoethanol). Protein concentrations were determined using a Qubit fluorometer. The protein extract (3.5 μg equivalents per lane) was subjected to SDS-polyacrylamide gel electrophoresis in a 10–20% gradient gel (ATTO, Tokyo, Japan) and then transferred onto a polyvinylidene difluoride (PVDF) membrane. After blocking with PVDF Blocking Reagent for Can Get Signal (TOYOBO, Osaka, Japan), the membrane was incubated with one of the following primary antibodies: rabbit polyclonal anti-human NPC2 antibody (ab186829, Abcam, Cambridge, UK), rabbit polyclonal anti-human IGFBP7 antibody (ab74169, Abcam), rabbit polyclonal anti-human ACTB antibody (GTX109639, GeneTex, Irvine, CA, USA), rabbit polyclonal anti-human GAPDH antibody (#2118, Cell Signaling Technology, Danvers, MA, USA), or mouse monoclonal anti-human THBS1 antibody (sc-59887, Santa Cruz Biotechnology, Dallas, TX, USA). After washing, the membrane was incubated with the following secondary antibody: horseradish peroxidase-labeled anti-rabbit immunoglobulin G (#7074, Cell Signaling Technology). Protein bands were visualized using an ECL Prime kit (GE Healthcare, Piscataway, NJ, USA) and were detected on a LAS-2000 image analyser (Fujifilm, Tokyo, Japan).

### Histological analysis

Portions of the surgical specimens from the TAAA patients and the NVDC patients were immediately fixed with 4% formalin overnight (neutral pH) and then embedded in paraffin, without dissection into three layers. For conventional histochemistry, paraffin-embedded tissues were cut into 3 μm-thick sections using a sledge microtome, mounted on slides, and processed with HE, MT and EVG stains to determine aortic wall morphology. Immunohistochemical (IHC) procedures were performed as previously described, using formalin-fixed, paraffin-embedded sections^50^. The following antibodies were used for IHC analyses: anti-NPC2 (ab186829, Abcam; 1:200) and anti-THBS1 (sc-59887, Santa Cruz Biotechnology; 1:200). Both of these antibodies were also used in western blotting analyses. For IGFBP7 analysis, a mouse monoclonal antibody against human IGFBP7 (sc-36529, Santa Cruz Biotechnology; 1:200) was used.

### Enzyme-linked immunosorbent assays

The concentrations of serum NPC2 and IGFBP7 and plasma THBS1 were measured using an NPC2 ELISA kit (Aviva Systems Biology, San Diego, CA, USA), an ELISA Kit for Insulin Like Growth Factor Binding Protein 7 (Cloud-Clone, Houston, TX, USA), and a Human Thrombospondin-1 Quantikine ELISA Kit (R&D Systems, Minneapolis, MN, USA), respectively, according to the respective manufacturer’s instructions.

### Statistical analysis

StatFlex Ver.6 (Artech, Osaka, Japan) and BellCurve for Excel (Social Survey Research Information, Tokyo, Japan) were used for statistical analyses. Welch’s t-test was used for comparisons of two groups and the Steel-Dwass test was used for comparisons of three or more groups. In these analyses, a p-value less than 0.05 was considered significant. ROC analysis was performed to evaluate the capacity of biomarker candidates to discriminate between HCs and atherosclerotic AA patients (TAAA and AAAA). For the combined analysis of biomarker candidates, discriminant analysis was first performed using their concentration data and the resulting discriminant score was subjected to ROC analysis.

## Supplementary information

Supplementary Information.
