# Publisher Correction: Discovery of novel biomarkers for atherosclerotic aortic aneurysm through proteomics-based assessment of disease progression

**DOI:** 10.1038/s41598-020-67561-x

**Published:** 2020-06-23

**Authors:** Hiroaki Yagi, Mitsuhiro Nishigori, Yusuke Murakami, Tsukasa Osaki, Sayaka Muto, Yutaka Iba, Kenji Minatoya, Yoshihiko Ikeda, Hatsue Ishibashi-Ueda, Takayuki Morisaki, Hitoshi Ogino, Hiroshi Tanaka, Hiroaki Sasaki, Hitoshi Matsuda, Naoto Minamino

**Affiliations:** 1 Department of Molecular Pharmacology, National Cerebral and Cardiovascular Center Research Institute, Suita, Osaka Japan; 2 Omics Research Center, National Cerebral and Cardiovascular Center, Suita, Osaka Japan; 3 Department of Vascular Surgery, National Cerebral and Cardiovascular Center, Suita, Osaka Japan; 4 Department of Pathology, National Cerebral and Cardiovascular Center, Suita, Osaka Japan; 5 Department of Bioscience and Genetics, National Cerebral and Cardiovascular Center Research Institute, Suita, Osaka Japan

Correction to: *Scientific Reports* 10.1038/s41598-020-63229-8, published online 14 April 2020

In Fig. 3B, incorrect protein names for each row are displayed. The correct Fig. 3B appears below as Fig. 1.

**Figure 1:**
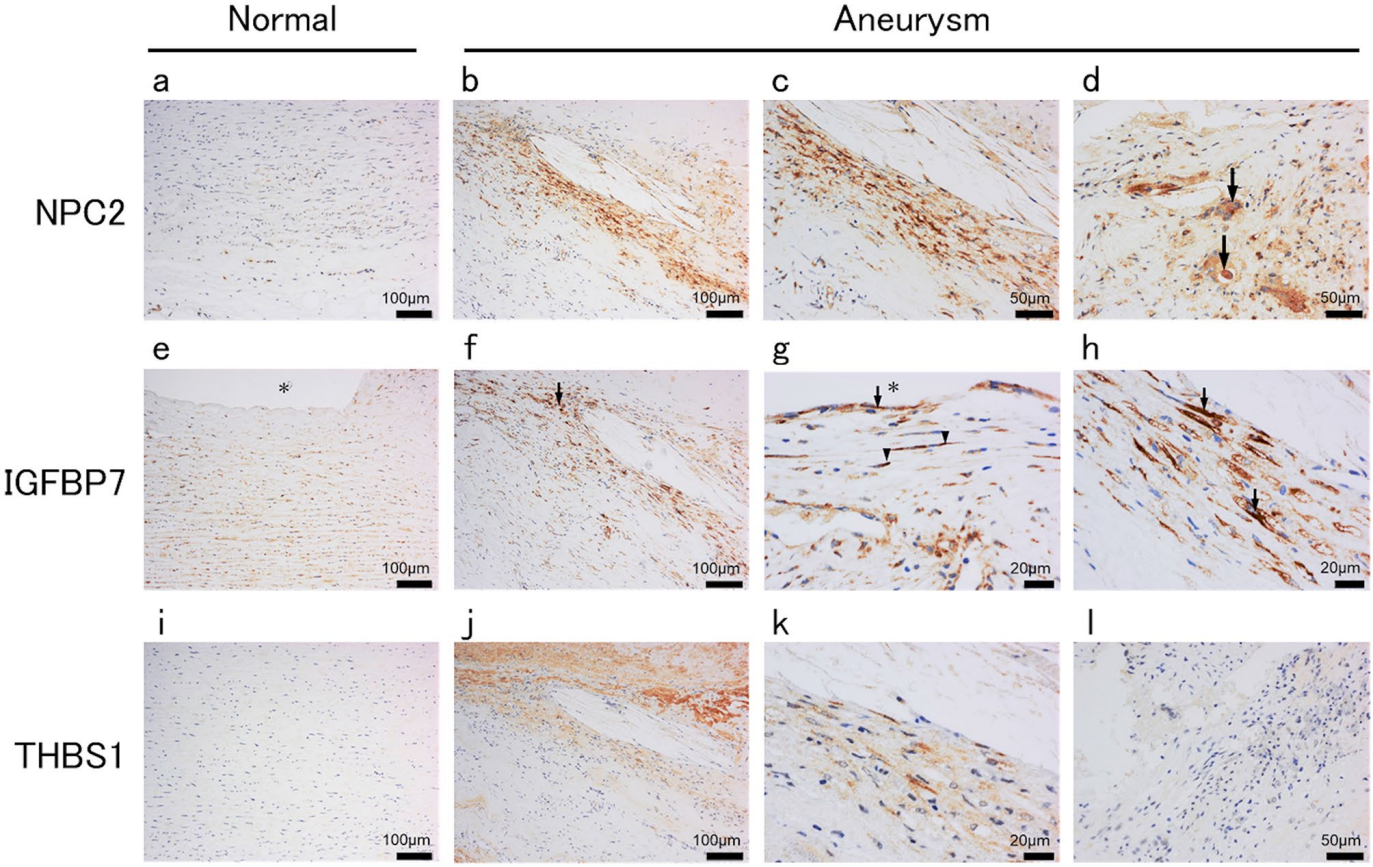
.

