# Supplementary material for: Discovery of novel biomarkers for atherosclerotic aortic aneurysm through proteomics-based assessment of disease progression
Source: Sci Rep. 2020 Apr 14;10:6429. doi: 10.1038/s41598-020-63229-8 (PMC7156426; doi:10.1038/s41598-020-63229-8)
Supplement: Supplementary file 1 — Supplementary Information. [file 41598_2020_63229_MOESM1_ESM.docx]

Supplementary information

Discovery of novel biomarkers for atherosclerotic aortic aneurysm

through proteomics-based assessment of disease progression

Hiroaki Yagi, Mitsuhiro Nishigori, Yusuke Murakami, Tsukasa Osaki, Sayaka Muto, Yutaka Iba, Kenji Minatoya, Yoshihiko Ikeda,

Hatsue Ishibashi-Ueda, Takayuki Morisaki, Hitoshi Ogino, Hiroshi Tanaka, Hiroaki Sasaki, Hitoshi Matsuda, and Naoto Minamino

Supplementary Figures, Tables, and Methods


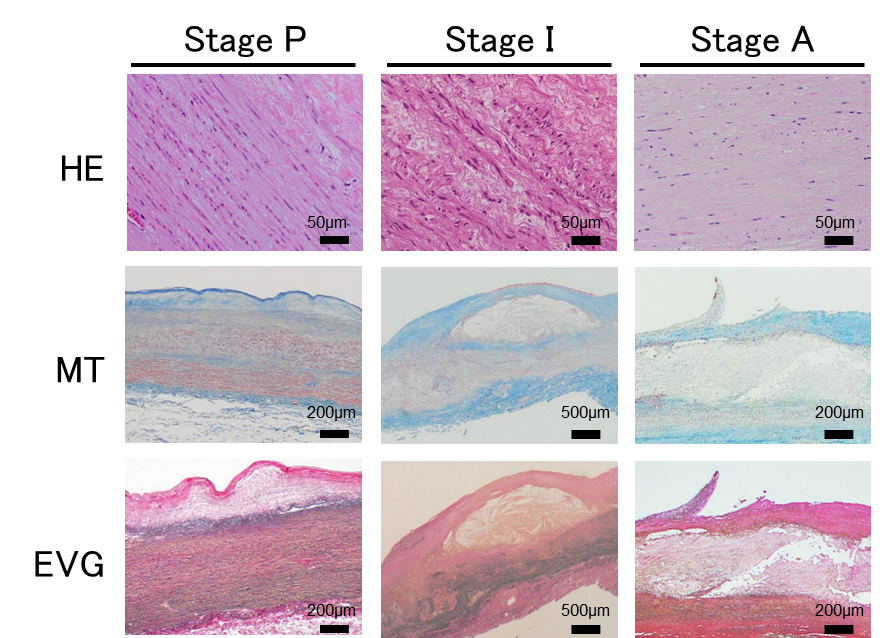


**Supplementary Figure 1. HE, MT, and EVG staining in serial sections of TAAA.**

Histopathological analysis of TAAA tissues at different disease progression stages. Upper: High magnification HE staining images. Middle: MT staining images. Lower: EVG staining images.


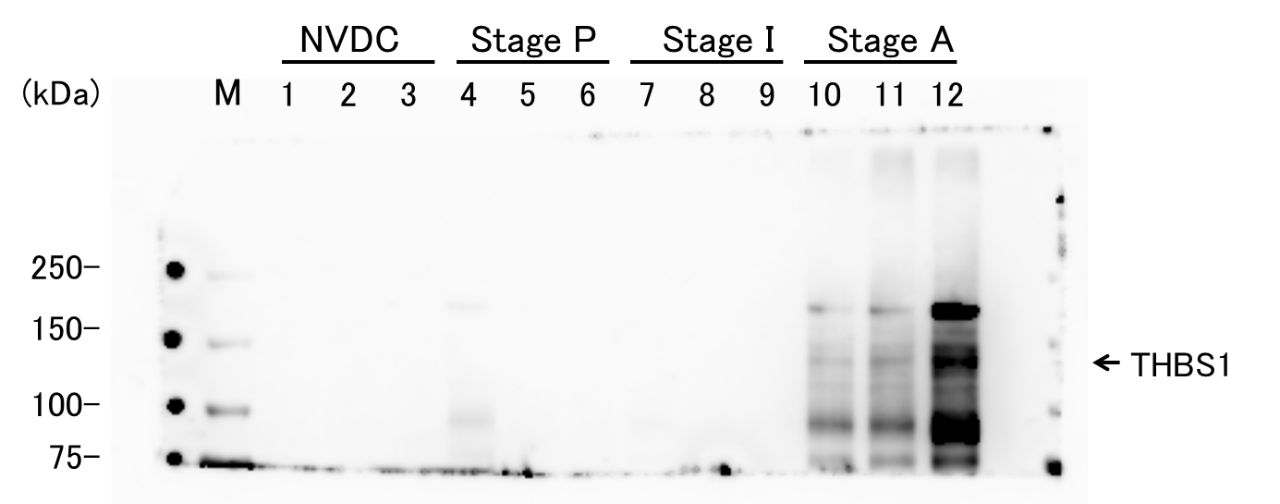
A


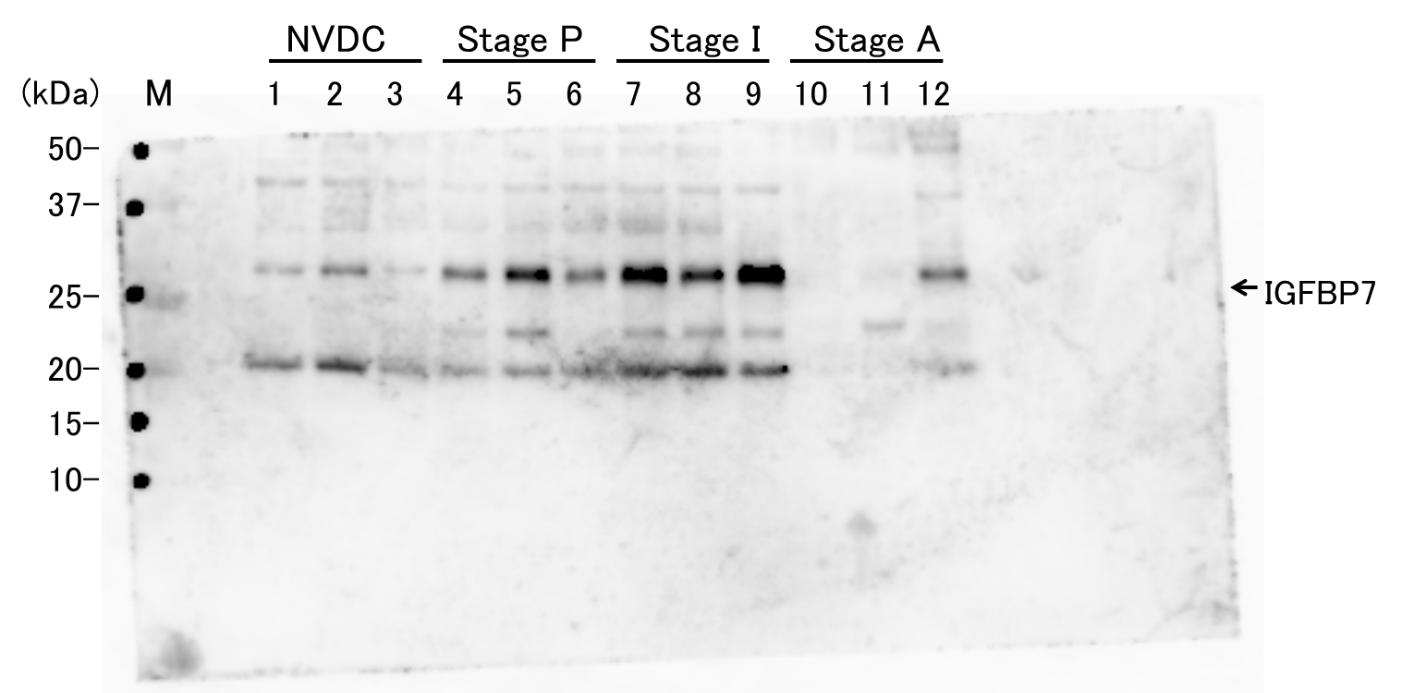
B


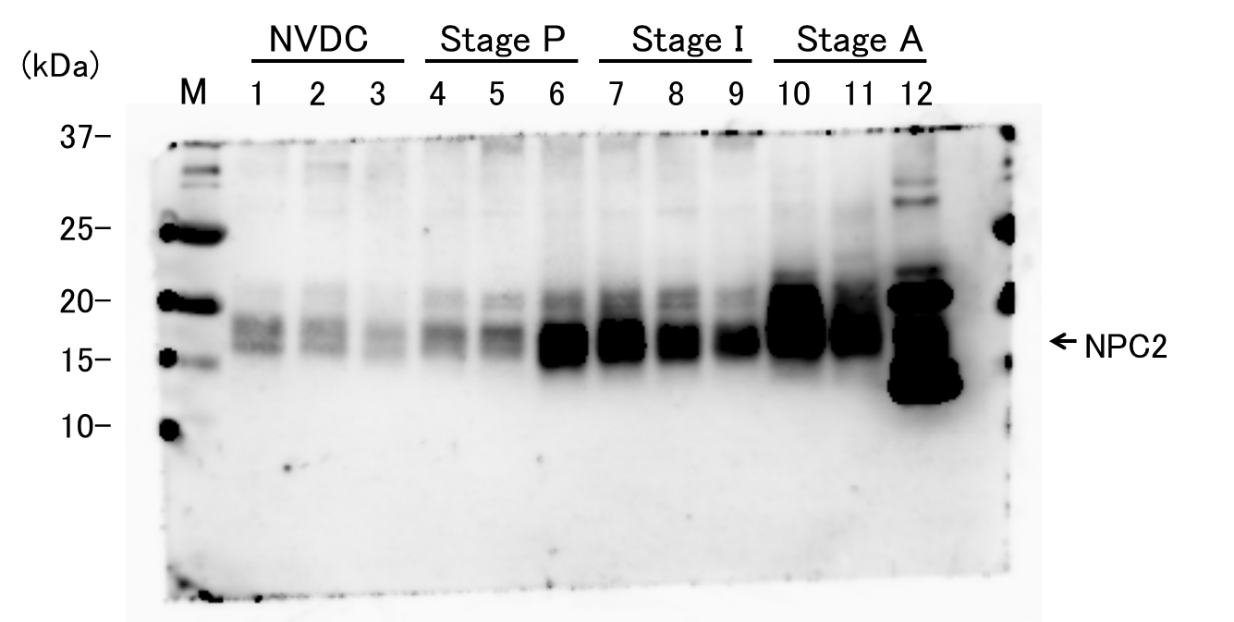
C


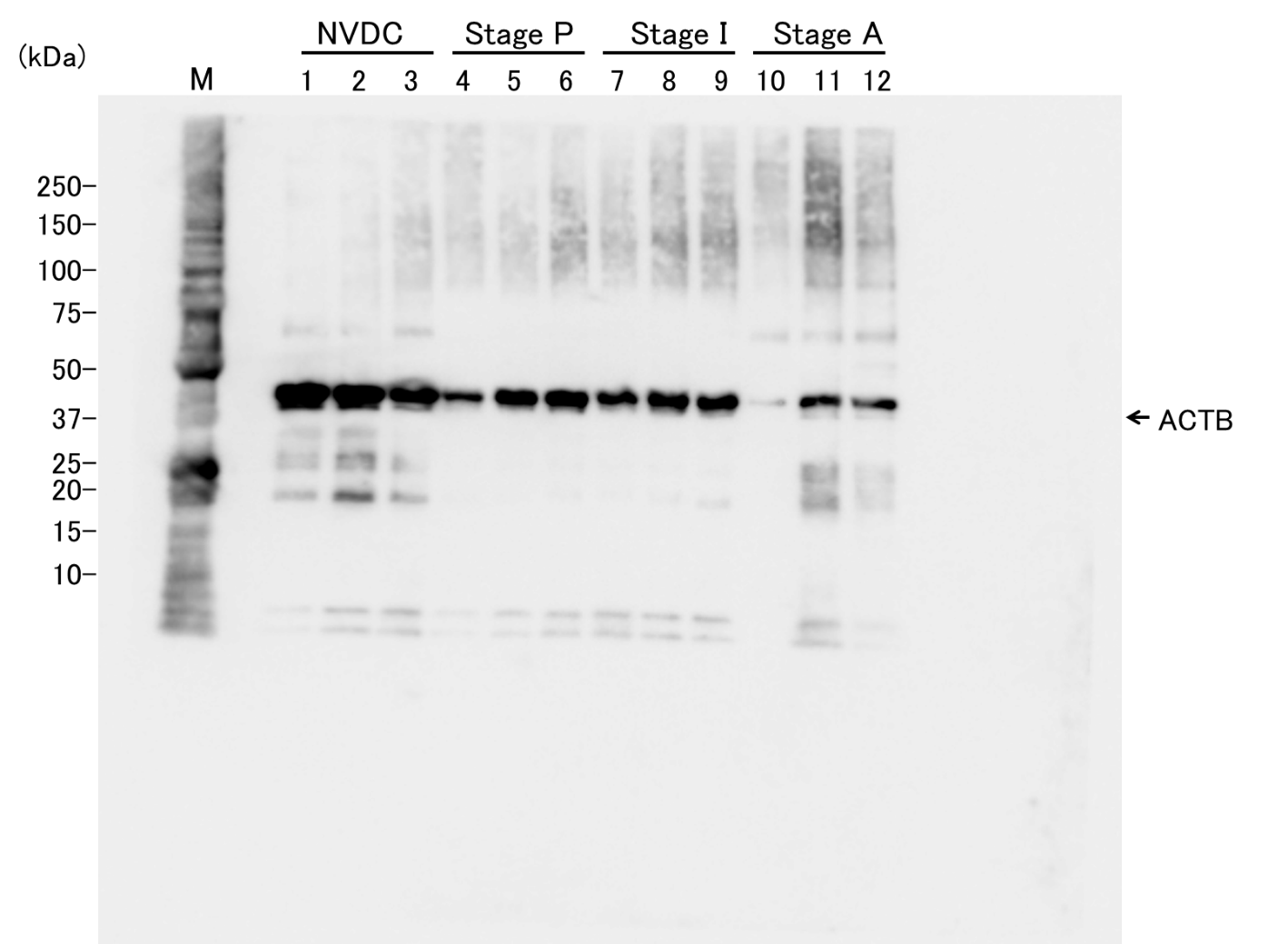
D

**Supplemental Figure 2. Whole images of western blotting analysis of NPC2, IGFBP7, and THBS1 in Fig. 3A**

(A) THBS1, (B) IGFBP7, (C) NPC2, and (D) ACTB levels in aortic tissues of NVDCs and TAAA patients are shown. The predicted positions of THBS1, IGFBP7, NPC2, and ACTB are indicated by arrows on the right side, while the positions of molecular size markers (lane M) and their molecular masses in kDa on the left side. (A) is a half gel image of a high molecular weight side, (B) and (C) are half gel images of low molecular weight sides, and (D) is a total gel image. Minor bands of 20.5 kDa in (B) are considered as proteolytic products of IGFBP7. The other information is the same as in the legend of Fig. 3A.


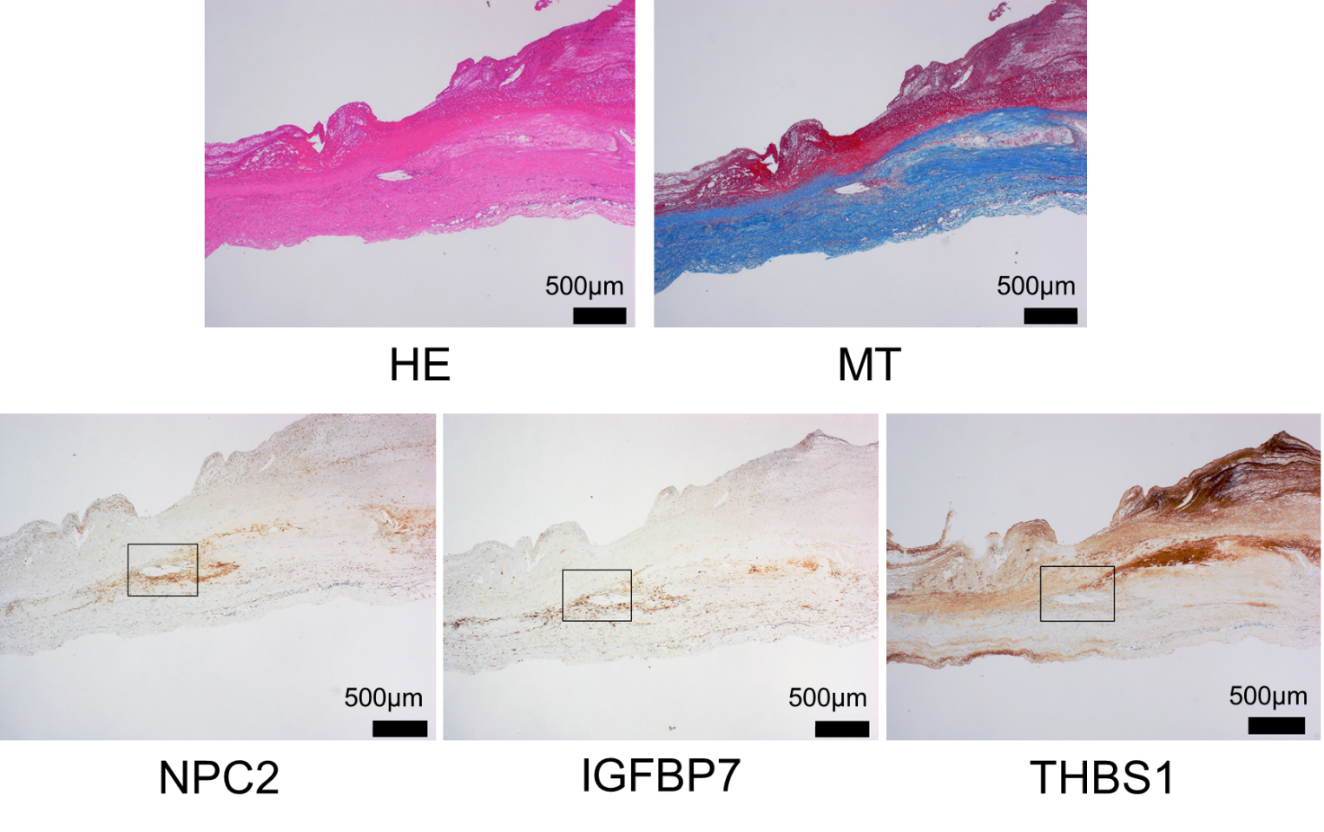


**Supplementary Figure 3. HE and MT staining and immunohistochemical analyses of NPC2, IGFBP7, and THBS1 in serial sections of TAAA.**

Upper: Low magnification HE and MT staining of TAAA. Lower: Low magnification immunohistochemical analysis of NPC2, IGFBP7, and THBS1. Boxes indicate the regions used for high magnification images in Figure 3B [b, f, and j]. HE, hematoxylin and eosin; MT, Masson’s trichrome; TAAA, thoracic atherosclerotic aortic aneurysm.

| GO number | GO term |
| --- | --- |
| GO:0032368 | Regulation of lipid transport |
| GO:0033700 | Phospholipid efflux |
| GO:0032374 | Regulation of cholesterol transport |
| GO:0032371 | Regulation of sterol transport |
| GO:0034377 | Plasma lipoprotein particle assembly |
| GO:0065005 | Protein-lipid complex assembly |
| GO:0030301 | Cholesterol transport |
| GO:0015918 | Sterol transport |
| GO:0051055 | Negative regulation of lipid biosynthetic process |
| GO:0010876 | Lipid localization |
| GO:0045833 | Negative regulation of lipid metabolic process |
| GO:0043691 | Reverse cholesterol transport |
| GO:0034381 | Lipoprotein particle clearance |
| GO:0010873 | Positive regulation of cholesterol esterification |
| GO:0006869 | Lipid transport |
| GO:0015914 | Phospholipid transport |
| GO:0010872 | Regulation of cholesterol esterification |
| GO:0033344 | Cholesterol efflux |
| GO:0019216 | Regulation of lipid metabolic process |
| GO:0010903 | Negative regulation of very-low-density lipoprotein particle remodeling |
| GO:0034375 | High-density lipoprotein particle remodeling |
| GO:0046890 | Regulation of lipid biosynthetic process |
| GO:0001568 | Blood vessel development |
| GO:0001944 | Vasculature development |
| GO:0032369 | Negative regulation of lipid transport |
| GO:0001936 | Regulation of endothelial cell proliferation |

**Supplementary Table 1. Atherosclerosis- and blood vessel-related GO terms enriched by GO analysis of proteins differentially expressed between proteomics-based progression stages**

**Supplementary Table 2. Number of aortic tissue samples in each category of morphological classification system and proteomics-based progression staging system**

|  | | Proteomics-based progression staging | | |
| --- | --- | --- | --- | --- |
|  |  | Stage P | Stage I | Stage A |
| Morphological classification | Group N | 20 | 3 | 0 |
|  | Group D | 13 | 6 | 10 |

The number in Group N was smaller than the number in Group D, because amounts of normal areas excised in six cases were not sufficient for proteome analysis.

**Supplementary Methods**

**Protein identification and** **quantification procedure in the proteome analysis**

1. Criteria for identification of trypsin-digested peptides in Mascot search

1.1. The parameter sets for MS/MS ion search were shown below;

*Database: SwissProt*

*Enzyme: Trypsin*

*Miss cleavages allowed: Up to one*

*Variable modification: Methionine oxidation*

*Peptide tolerance: 20 ppm*

*MS/MS tolerance: 0.05 Da*

1.2. The peptides identified with an expectation value p<0.05 were considered as reliable.

1.3. If multiple peptides with high sequence similarity were selected for the same peptide peak, the peptide sequence is not determined unambiguously and is excluded from the identified peptide list.

1.4. PeptideProphet^1^ analysis was also performed and the results were used to select peptide peaks for the protein quantification, as described in 3.2.

2. Quantification of tryptic peptides by the non-labeling method

2.1. MS peak intensities did not always correlate with absolute amounts of peptides. However, if samples of the same tissue were processed by the fixed method, the peptide peak intensity ratio of the same peptide is expected to correlate with the relative abundance of the peptides. This is the principle of the non-labeling quantitative proteomic analysis method.

2.2. In the 2DICAL software, the MS peaks of the same peptides were adjusted by the specific program and superimposed, and the average peak intensity was calculated and compared between the sample groups.

2.3. If the same peptide was identified as various charged forms (2+, 3+, 4+), the highest peak is selected for the quantitative analysis.

3. Selection method of tryptic peptide peaks for protein quantification

3.1. The protein levels in the target tissue were evaluated by using multiple peptide peak data from each protein.

3.2. The peptide peak data are filtered by the following rule;

*a. If the weight value in PeptideProphet analysis was less than 0.5, the peptide is excluded.*

*b. If multiple peptides showed nearly equal values in both m/z and retention time (within 0.05Da and 0.6 min each), these peptides are excluded except the value of number of sibling peptides (NSP)-adjusted probability in the PeptideProphet analysis was more than 0.8.*

*c. The peptides including methionine are excluded because methionine residue is easily oxidized during the sample preparation.*

*d. Peptides generated by miss cleavages of trypsin/lysyl endopeptidase are excluded.*

*e. Peptides with the low peak intensity (mean intensity of the group is less than 500) are excluded.*

*f. Peptides of which peaks are detected in less than 90 % of samples are excluded.*

3.3. Among the peptides satisfying the above criteria, the average of three peptides with highest intensity are used for the protein quantification.

**Reference:**

1 Shteynberg, D. *et al.* iProphet: multi-level integrative analysis of shotgun proteomic data improves peptide and protein identification rates and error estimates. *Mol Cell Proteomics* **10**, M111.007690 (2011). doi:10.1074/mcp.M111.007690
